# Supplementary material for: Two Decades Later: Long-Term Multisystem Sequelae and Subclinical Organ Dysfunction in Sudan Ebola Virus (SUDV) Survivors of the 2000 Outbreak
Source: Viruses. 2025 Oct 23;17(11):1410. doi: 10.3390/v17111410 (PMC12656871; doi:10.3390/v17111410)
Supplement: Supplementary file 1 [file viruses-17-01410-s001.zip › viruses-3875661-supplementary.pdf]

COALITION FOR EPIDEMIC PREPAREDNESS INNOVATIONS (CEPI)

Long-term Health and Socio-Economic Impact of Ebola Virus Disease (EVD) and Marburg Among Survivors in Uganda

Section 1: Participant Information

1.1 **PID-** Participant ID: ______________________________

1.2 **DOA-** Date of Assessment: __ __/__ __ __/__ __ __ __ (DD / MMM /YY)

1.3 **INT_ID-** Interviewer Name/ID: ____________________

1.4 **GRP-** Study Group:

1 ☐ EVD Survivor **2** ☐ Marburg Survivor **3** ☐ Unexposed Uninfected (**UU**)

4 ☐ Exposed Uninfected Contact (**EU**) **5** ☐ Previously Vaccinated (**PV**)

Section 2: Vital Signs

2.1 **BP-** Blood Pressure: ___________ mmHg

2.2 **HR-** Heart Rate: ___________ bpm

2.3 **RR-** Respiratory Rate: ___________ breaths/min

2.4 **TMP-** Temperature: ___________ °C

ANTHR-Anthropometry

2.5 **HT-** Height: _______ cm

2.6 **WT-** Weight: _______ kg

2.7 **BMI-** Body Mass Index (BMI): _______ kg/m²

Section 3: Demographic Information

3.1 **AGE-** Age: __________ (years)

3.2 **DOB-** Date of Birth: __ __/__ __ __/__ __ __ ___

3.3 **RES-** Residence (District): ______________________

3.4 **GND-** Gender: **1** ☐ Male **2** ☐ Female

3.5 **MAR-** Marital Status: **1** ☐ Single **2** ☐ Married **3** ☐ Widowed **4** ☐ Divorced

3.6 **EDU-** Educational Level:

1 ☐ No formal education

2 ☐ Primary education

3 ☐ Secondary education

4 ☐ College/University

3.7 **EMP-** Employment Status: (Income sources formal employment, informal, farming, etc.)

1 ☐ Employed (full-time)

2 ☐ Employed (part-time)

3 ☐ Self-employed

4 ☐ Unemployed

5 ☐ Retired

6 ☐ Student

3.8 **OCC-** Occupation: ____________________________________

3.9 **HSZ-**Household Size:

1 ☐ Less than 5

2 ☐ 5-10

3 ☐ Greater than 10

3.10 **NRO-**Number of rooms in the house: ______________________
3.11 **HHD-**Number of people living per room______________ (Household Density)
3.12 **DWE-**Type of dwelling:
1 ☐ Permanent
2 ☐ Semi-permanent
3 ☐ Temporary
3.13 **CLW**- Water Source: **1** ☐ Piped water **2** ☐ Borehole **3** ☐ Well **4** ☐ River
3.14 **COO** – Cooking Fuel Used**: 1** ☐ Firewood **2** ☐ Charcoal **3** ☐ Gas **4** ☐ Electricity
3.15 **OHI**-Ownership of household items: **1**☐ radio 2 ☐ television **3** ☐refrigerator **4** ☐ mobile phone
3.16 **ELE**- Access to electricity: **1** ☐ Yes **2** ☐ No
3.17 **INT**- Access to Internet: **1** ☐ Yes **2** ☐ No
3.18 **DIG**- Access to digital devices: (smartphone, computer):
1 ☐ Yes **2** ☐ No

Section 4: Ebola Virus/Marburg Disease (EVD/MVD) History

4.1 EVD/MVD History:
EVD_MAR_DIAG- Diagnosis:
1 ☐ Confirmed EVD **2** ☐ Confirmed Marburg
DIAG_DT- Date of Diagnosis: __ __/__ __ __/__ __ __ __ *(DD / MMM /YY)*
REC_DT- Date of Recovery: __ __/__ __ __/__ __ __ __ *(DD / MMM /YY)*
4.2 **ADM**- Admitted for Treatment: **1** ☐ Yes **2** ☐ No
4.3 **HOS_NM**- Hospital/Clinic Name: _________________________
4.4 **DUR_HOSP**- Duration of Hospitalization (days): _______________
4.5 **TX_REC**-Treatments Received (check all that apply):
1 ☐ Antiviral medication (specify if known): _______________
2 ☐ IV fluids
3 ☐ Respiratory support (e.g., oxygen)
4 ☐ Blood transfusion
5 ☐ Nutritional support
6 ☐ None
7 ☐ Other (specify): __________________________________

Symptoms during outbreak

4.6 **SYS_ON**- Initial symptoms at onset (tick all applicable)
1 ☐ Fever
2 ☐ Headache
3 ☐ Muscle pain
4 ☐ Vomiting
5 ☐ Diarrhoea
6 ☐ Abdominal pain
7 ☐ Other (specify): __________________________________

4.7 **SYS_PR-** Progression of symptoms (tick all applicable)

1 ☐ Fever
2 ☐ Headache
3 ☐ Muscle pain
4 ☐ Vomiting
5 ☐ Diarrhoea
6 ☐ Abdominal pain
7 ☐ Other (specify): ___________________________________

4.8 **SYP_SV-** Severe symptoms, presence of any high-risk symptoms like bleeding, organ failure, neurologic symptoms (tick all applicable)

1 ☐ Bleeding
2 ☐ Confusion
3 ☐ Difficulty breathing
4 ☐ Other (specify): ___________________________________

Persistent Symptoms Post-EVD/MVD Recovery

4.9 **FAT- Fatigue: 1** ☐ Yes **2** ☐ No

4.10 **FAT_DUR-**Duration: ______________________ *(Weeks, Months)*	4.11 **FAT_IA-** Impact on daily activities:<br>**1** ☐ None<br>**2** ☐ Mild<br>**3** ☐ Moderate<br>**4** ☐ Severe

4.12 **JP-** Joint Pain: **1** ☐ Yes **2** ☐ No

4.13 **JP_LOC-** Location (check all that apply):<br>**1** ☐ Knees<br>**2** ☐ Elbows<br>**3** ☐ Wrists<br>**4** ☐ Hips<br>**5** ☐ Other (specify): _______________________<br><br>4.14 **JP_DUR-** Duration: ______________________ *(Weeks, Months)*	4.15 **JP_IA-** Impact on daily activities:<br>**1** ☐ None<br>**2** ☐ Mild<br>**3** ☐ Moderate<br>**4** ☐ Severe

4.16 **MWP-** Muscle Weakness/Pain: **1** ☐ Yes **2** ☐ No

4.17 **MWP_AA-** Affected Areas (specify): ____________________________________<br><br>4.18 **MWP_DUR-** Duration: ____________________ *(Weeks, Months)*	4.19 **MWP_IA-** Impact on daily activities:<br>**1** ☐ None<br>**2** ☐ Mild<br>**3** ☐ Moderate<br>**4** ☐ Severe

4.20 **HD-**Headaches: **1** ☐ Yes **2** ☐ No

4.21 **HD_T-** Type (e.g., tension, migraine) ____________________<br>4.22 **HD_F-** Frequency (e.g., daily, weekly) ____________________<br>4.23 **HD_DUR-** Duration: ____________________ *(Weeks, Months)*	4.24 **HD_IA-** Impact on daily activities:<br>**1** ☐ None<br>**2** ☐ Mild<br>**3** ☐ Moderate<br>**4** ☐ Severe

4.25 **VP-**Vision Problems: **1** ☐ Yes **2** ☐ No

4.26 **VP_TY-** Type (check all that apply)<br>**1** ☐ Blurriness<br>**2** ☐ Sensitivity to light<br>**3** ☐ Other (specify): ____________________<br>4.27 **VP_DUR-** Duration: ____________________ *(Weeks, Months)*	4.28 **VP_IA-** Impact on daily activities:<br>**1** ☐ None<br>**2** ☐ Mild<br>**3** ☐ Moderate<br>**4** ☐ Severe

4.29 **HI-** Hearing Issues: **1** ☐ Yes **2** ☐ No

4.30 **HI_TY-** Type (check all that apply)<br>**1** ☐ Hearing loss<br>**2** ☐ Tinnitus (ringing in ears)<br>**3** ☐ Other (specify): ____________________<br>4.31 **HI_DUR-** Duration: ____________________ *(Weeks, Months)*	4.32 **HI_IA-** Impact on daily activities:<br>**1** ☐ None<br>**2** ☐ Mild<br>**3** ☐ Moderate<br>**4** ☐ Severe

4.33 **NS-**Neurological Symptoms: **1** ☐ Yes **2** ☐ No

4.34 **NS_TY-** Type (check all that apply)<br>**1** ☐ Numbness or tingling (where): __________<br>**2** ☐ Memory problems<br>**3** ☐ Difficulty concentrating<br>**4** ☐ Confusion<br>**5** ☐ Other (specify): ____________________<br>4.35 **NS_DUR-** Duration: ____________________ *(Weeks, Months)*	4.36 **NS_IA-** Impact on daily activities:<br>**1** ☐ None<br>**2** ☐ Mild<br>**3** ☐ Moderate<br>**4** ☐ Severe

4.37 **GI-** Gastrointestinal Issues: **1** ☐ Yes **2** ☐ No

4.38 **GI_TY-** Type (check all that apply)<br>**1** ☐ Nausea<br>**2** ☐ Diarrhea<br>**3** ☐ Abdominal pain<br>**4** ☐ Loss of appetite<br>**5** ☐ Other (specify): ____________________<br>4.39 **GI_DUR-** Duration: ____________________<br>*(Weeks, Months)*	4.40 **GI_IA-** Impact on daily activities:<br>**1** ☐ None<br>**2** ☐ Mild<br>**3** ☐ Moderate<br>**4** ☐ Severe

4.41 **OS-**Other Symptoms: **1** ☐ Yes **2** ☐ No

Please specify any other persistent symptoms not listed above: ____________________<br>____________________<br>____________________<br>4.42 **OS_DUR-** Duration: ____________________<br>*(Weeks, Months)*	4.43 **OS_IA-** Impact on daily activities:<br>**1** ☐ None<br>**2** ☐ Mild<br>**3** ☐ Moderate<br>**4** ☐ Severe

Section 5: General Medical and Family History

5.1 **PMH-**Pre-existing Conditions (prior to EVD/MVD):

**1** ☐ Hypertension	**6** ☐ HIV/AIDS
**2** ☐ Diabetes	**7** ☐ Mental health issues
**3** ☐ Heart disease	**8** ☐ None
**4** ☐ Tuberculosis	**9** ☐ Other (specify): ____________________
**5** ☐ Visual impairment	

5.2 **PO_MH-**Post-existing Conditions (after EVD/MVD):

**1** ☐ Hypertension	**6** ☐ HIV/AIDS
**2** ☐ Diabetes	**7** ☐ Mental health issues
**3** ☐ Heart disease	**8** ☐ None
**4** ☐ Tuberculosis	**9** ☐ Other (specify): ____________________
**5** ☐ Visual impairment	

5.3 **FAM_HX-** Family History:

**1** ☐ Hypertension	**5** ☐ Mental health conditions
**2** ☐ Diabetes	**6** ☐ None
**3** ☐ Heart disease	**7** ☐ Other (specify):____________________
**4** ☐ Sickle cell disease	

5.4 **VAC-** Vaccination Status (list recent vaccinations):_______________________________

5.5 **ALL**-Allergies:

ALL_DRUG-Drug Allergies: _______________________________

ALL_ENV- Environmental Allergies: __________-_______________

5.6 **MEDS-** Current Medications:

**MED1**-Medication 1	
**MED2-** Medication 2	

Section 6: Physical Examination Findings

Pain and Musculoskeletal Findings:

6.1 **PPR-** Pain Presence: **1**☐ Yes **2**☐ No

6.2 **PLC**-Pain Location(s):

**1** ☐ Head<br>**2** ☐ Neck<br>**3** ☐ Shoulders	**4** ☐ Arms<br>**5** ☐ Lower back<br>**6** ☐ Joints (specify which): _____________

6.3 **PSV**-Pain Severity (0-10 scale): __________

6.4 **JSW**-Joint Swelling or Tenderness: **1** ☐ Yes **2** ☐ No

3 ☐ (specify locations if applicable): ________________, ________________

6.5 **HNE**-Head and Neck Examination:

**HDE**-**Head:**	**1** ☐ Normocephalic<br>**2** ☐ Signs of trauma or deformity
**EYE**-**Eyes:**	**1** ☐ Pupils Equal and reactive<br>**2** ☐ Nystagmus present<br>**3** ☐ Vision changes (specify): _______________________________
**EAE**-**Ears:**	**1** ☐ Clear canals<br>**2** ☐ Discharge present (specify): ____________________________
**NOE**-**Nose:**	**1** ☐ Patent<br>**2** ☐ Nasal congestion
**THE**-**Throat:**	**1** ☐ Mucosa moist<br>**2** ☐ Erythema or lesions present<br>**3** ☐ Tonsillar enlargement

6.6 **CVE-** Cardiovascular Examination:

**HR_S-**Heart Sounds:	**1** ☐ Regular<br>**2** ☐ Irregular (specify details): ______________________________
**P_PS-**Peripheral Pulses:	**1** ☐ Radial: Present and strong<br>**2** ☐ Dorsalis pedis: Present<br>**3** ☐ Posterior tibial: Present<br>**4** ☐ Weak or absent (specify): ______________________________
**EDM-** Edema**:**	**1** ☐ None<br>**2** ☐ Present (specify location and extent): ______________________

6.7 **REX-**Respiratory Examination:

**BR_S-Breath Sounds:**	**1** ☐ Clear bilaterally<br>**2** ☐ Wheezing<br>**3** ☐ Crackles<br>**4** ☐ Decreased air entry (specify side): _________________________
**COU-**Cough**:**	**1** ☐ Productive<br>**2** ☐ Non-productive<br>**3** ☐ Sputum: Color /consistency (specify): _______________________
**CH_WA-**Chest Wall:	**1** ☐ Symmetrical<br>**2** ☐ Abnormal movement (specify): ____________________________

6.8 **GE-**Gastrointestinal Examination:

**Abdomen:**	
**GE_IN-**Inspection:<br>**1** ☐ Normal fullness<br>**2** ☐ Distended<br>**3** ☐ Scaphoid<br>**4** ☐ Scars present (specify): ____________<br>**GE_PA-**Palpation:<br>**1** ☐ No tenderness<br>**2** ☐ Tenderness (specify location): __________<br>**3** ☐ Masses (specify): ____________________	**GE_BS-** Bowel Sounds:<br>**1** ☐ Normal<br>**2** ☐ Decreased<br>**3** ☐ Absent

6.9 **MSE-**Neurological Examination (Motor and Sensory Examination):

MSE_WP-Weakness or paralysis: **1** ☐ Yes **2** ☐ No

MSE_CI-Coordination issues: **1** ☐ Yes **2** ☐ No

MSE_SI-Sensory impairments: **1** ☐ Yes **2** ☐ No **3** ☐ Specify (e.g., numbness, tingling): ________________________

6.10 **SKE-**Skin Examination:

**SK_I-Integrity:**	**SK_R_Rash:**	**SK_HS-Hydration Status:**
**1** ☐ Intact<br>**2** ☐ Lesions (specify type and location): ______________	**1** ☐ Present (specify description):<br>_____________________<br>**2** ☐ Absent	**1** ☐ Well-hydrated<br>**2** ☐ Dehydrated (specify signs):<br>________________

6.11 **AOB-**Additional Observations:

**AOB_NF**-Any other notable findings:	**AOB_REC**- Recommendations for further evaluation or referral:

Section 7: Mental Health Assessment

7.1 Anxiety Symptoms (based on Hospital Anxiety and Depression Scale - HADS)
ANX-Total Anxiety Score: _____________
ANX_SC-Interpretation:
- **1** ☐ Normal
- **2** ☐ Mild
- **3** ☐ Moderate
- **4** ☐ Severe

7.2 Depression Symptoms (based on HADS)
DPR-Total Depression Score: ___________
DPR_SC-Interpretation:
- **1** ☐ Normal
- **2** ☐ Mild
- **3** ☐ Moderate
- **4** ☐ Severe

Section 8: Healthcare Utilization and Access

8.1 **POST_REC**-Frequency of Healthcare Visits Post-Recovery
- **1** ☐ Weekly
- **2** ☐ Monthly
- **3** ☐ Quarterly
- **4** ☐ Rarely

8.2 **BAR_HC**-Barriers to Accessing Healthcare (Tick all that apply)
- **1** ☐ Financial constraints
- **2** ☐ Transportation issues
- **3** ☐ Lack of specialized care
- **4** ☐ Social stigma
- **5** ☐ Other (Specify): ___________________________

Section 9: Social Support and Stigma

9.1 **PER_STI**-Perceived Stigma

Have you experienced stigma or discrimination due to your EVD/MVD status?

1 ☐ Yes **2** ☐ No

9.2 **SSP**-Social Support

Do you have family or community support in managing your health post-EVD/MVD?

1 ☐ Yes **2** ☐ No

9.3 Coping Mechanisms

COM- What coping mechanisms do you use? (Tick all that apply)

1 ☐ Family support

2 ☐ Community support

3 ☐ Religious practices

4 ☐ Physical activities

5 ☐ Counselling

6 ☐ Other (Specify): ______________________________

Section 10: Laboratory and Radiological Findings

10.1 **LAB** - Laboratory Tests:

Complete Blood Count (CBC):	**1** ☐ Yes **2** ☐ No
Liver Function Tests	**1** ☐ Yes **2** ☐ No
Renal Function Tests (Creatinine/BUN):	**1** ☐ Yes **2** ☐ No
Electrolytes (Na, K, Cl):	**1** ☐ Yes **2** ☐ No
Inflammatory Markers (CRP):	**1** ☐ Yes **2** ☐ No
Random Blood Sugar:	

Additional Observations and Notes

10.2 **Participant Remarks**:
10.3 **Other Relevant Findings or Observations**:
10.4 **Interviewer Comments:**
